# A mobile delivered self-exercise program for female farmers

**DOI:** 10.1097/MD.0000000000023624

**Published:** 2020-12-24

**Authors:** Sora Baek, Gowun Kim, Hee-won Park

**Affiliations:** a Center for Farmers’ Safety and Health and Department of Rehabilitation Medicine, Kangwon National University Hospital; b Department of Rehabilitation Medicine, School of Medicine, Kangwon National University, Chuncheon-si, Gangwon-do, Chuncheon, Republic of Korea.

**Keywords:** eHealth program, farmer, mobile application, musculoskeletal pain, rural area, self-exercise, tailored exercise program

## Abstract

**Introduction:**

Female farmers commonly experience musculoskeletal pain in the back, knee, and shoulder. Despite their obvious advantages in reducing musculoskeletal pain, face-to-face exercise programs are limited by geographical and physical barriers. Thus, we decided to introduce eHealth technology to farmers’ musculoskeletal health care. Using a mobile application (app), we aim to provide a tailored self-exercise program for shoulder, knee, and back pain in female farmers in rural areas after a musculoskeletal health check-up.

**Methods:**

This study is planned as 2 randomized control studies (MObile Delivered self-Exercise [MODE] phase I and phase II). We plan to recruit 200 female farmers aged 41 to 70 years. Initially, the shoulders, knees, and low back will be evaluated to provide individualized exercise programs. In MODE-I (single-blinded: evaluator), the subjects will be randomly allocated to experimental (n = 100) and control (n = 100) groups using a computer-generated sequence. Both groups will perform a 3-month self-exercise using a smartphone app or physical education data (booklets), respectively. Outcomes including exercise completion will be assessed at 3 months. In MODE-II, after subject random allocation, the experimental and control groups will perform exercise using a smartphone app with and without real-time feedback, respectively. Every 3 months, the level of the exercise program will be evaluated and the difficulty level will be adapted accordingly. After MODE-II is completed, all subjects will undergo close-out assessment.

**Discussion:**

This will be the first attempt to compare methods using booklets and apps to identify effective ways of providing personalized self-exercise programs according to musculoskeletal health stages by evaluating female farmers (MODE-I). This will help clarify whether the mobile app is effective for self-exercise compared to a conventional booklet. The MODE-II study will help to assess the effect of providing feedback through the mobile app. Finally, we will evaluate musculoskeletal health according to the degree of participation over 12 months to confirm the effect of self-exercise. Our study should aid in managing musculoskeletal health for farmers living in rural areas and help promote health in the “untact” era.

**Trial registration:**

Clinical Research Information Service of the Korean National Institutes of Health (KCT0005245). Registered July 17, 2020.

## 1 Introduction

Farmers commonly experience musculoskeletal pain in the back, knee, and shoulder, and female farmers are particularly more vulnerable to musculoskeletal disorders than men.^[1]^ Therefore, the Ministry of Agriculture, Food, and Rural Affairs in Korea plans to conduct musculoskeletal health checks for female farmers in the near future. The government-led health examinations primarily aim to identify people with musculoskeletal disorders.

Musculoskeletal disorders are very common and tend to worsen over time without proper managements. Therefore, a comprehensive management for preventing diseases and maintaining function as well as symptom improvement is considerably important. Exercise programs are very effective in reducing musculoskeletal pain.^[2]^ To date, exercise programs have been conducted face-to-face. Despite their obvious advantages in reducing musculoskeletal pain, face-to-face education and exercise programs are limited in their overall implementation,^[3]^ and it is expected that there will be limitations in conducting face-to-face exercise programs, which are suitable for candidates at the national level.

A health check-up project will be launched to evaluate the musculoskeletal health of female farmers. Rural areas have a large area and a low population density. In areas with low population densities, human resource limitations are expected to be maintained in community-based centers of exercise. To overcome geographical and physical barriers, we decided to introduce eHealth technology to farmers’ musculoskeletal health care.

By developing a self-exercise provided through a mobile application (app), we can provide female farmers living in rural areas with a tailored exercise program for shoulder, knee, and back pain after a baseline assessment. The mobile app is developed without feedback (feedback-) and with real-time feedback (feedback+). In phase I of this study, the effect of the feedback- app will be compared with the classic booklet with respect to self-exercise, and in phase II, the effects of the feedback+ and feedback- apps on self-exercise will be compared.

Our hypotheses are that the feedback- app-delivered self-exercise program will result in higher completion rates of 3-month self-exercise compared to the booklet-delivered self-exercise program, and that the feedback+ app-delivered exercise program will result in higher completion rates of 9-month self-exercise compared to the feedback- app-delivered exercise program.

## 2 Methods/design

### 2.1 Study design

**Figure 1:**
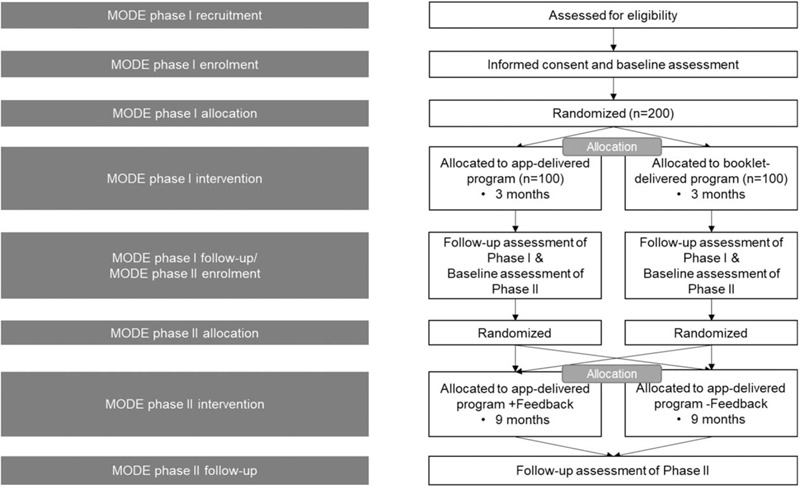
Consolidated Standards of Reporting Trials (CONSORT) flow diagram including participants’ recruitment, eligibility, screening, randomization, and outcome assessments of MODE phase I and II.

This study is planned as 2 randomized control trials. The phase I and II trials of the MObile Delivered self-Exercise (MODE) study will be randomized, prospective, single-blind (evaluator), and include active comparator controlled trials to test the effect of an app-delivered self-exercise program on the facilitation of self-exercise, musculoskeletal pain, and health behavior in female farmers aged 41 to 70 years. This study has been approved and conforms to the Declaration of Helsinki. This study was designed according to the CONsolidated Standards Of Reporting Trials (CONSORT) statement^[4]^ and is reported according to the Standard Protocol Items: Recommendations for Interventional Trials (SPIRIT) statement.^[5]^ The schedule of trial enrolment and assessments is shown in Table 1. The trial flow chart is shown in Figure 1.

**Table 1:** The schedule of trial enrolment and assessments.

	Study Period								
Assessment	Enrolment	Allocation	MODE I		MODE II				Close-out
Time point	-t_1_	0	t_1_	t_2_	t_3_	t_4_	t_5_	t_5_	t_6_
Enrolment:									
Eligibility screen	X								
Informed consent	X								
Allocation		X			X				
Interventions:									
Tool usage education			X		X				
App-delivered exercise group				X		X	X	X	
Booklet-delivered exercise group				X					
Assessment:									
Demographics		X							X
Agricultural information		X							X
Medical history		X							
Current medication		X							X
Smartphone usability		X							
Participation in self-exercise				X		X	X	X	
Satisfaction with self-exercise				X		X	X	X	
Depression (PHQ-9)		X		X					X
Shoulder disability (SPADI)		X		X					X
Hip and knee disability (WOMAC)		X		X					X
Back disability (ODI)		X		X					X
Vital sign		X							X
Anthropometric measurement		X		X					X
Bioimpedance analysis		X		X					X
x-Ray		X							X
Bone density DEXA		X							X
Trunk extension strength		X							X
Musculoskeletal physical examination		X							X
Hand grip strength		X							X
Balance test (TUG and BBS)		X							X
Pulmonary function test		X							X
Blood chemistry		X							X

### 2.2 Trial status

The enrolment of participants is currently ongoing at the time of submission study. Recruitment began on July 7, 2020.

### 2.3 Participants

The subjects will be recruited through women's farmers’ organizations in connection with the Gangwondo Agricultural Research and Extension Services. Subjects’ eligibility for inclusion will be determined during the screening process. If eligible, subjects will be assigned to 1 of the 2 groups, experimental or control groups, according to the randomization method. Personal data will be numerically coded and stored in a separate database. All study information will be provided to all participants.

### 2.4 Inclusion criteria

The inclusion criteria are as follows: women who cultivate crops on farmland exceeding 1000 m^2^ or engage in agriculture for ≥90 days in 1 year; women who cultivate crops by installing facilities >330 m^2^, such as a fixed greenhouses, mushroom barns, or vinyl-greenhouses; women who breed ≥2 large livestock, ≥10 medium livestock, ≥100 small livestock, ≥1000 birds or ≥10 beehives, or who are engaged in the livestock industry for ≥120 days or more; women with annual sales of agricultural products exceeding 1.2 million won accruing from agricultural management; and smartphone users.

### 2.5 Exclusion criteria

The exclusion criteria are as follows: women with trauma and surgical history of shoulders, knees, and low back within the last 6 months; subjects with systemic diseases that cause chronic back pain (eg, cancer, infection, and so on); those whose limbs (part or whole) are amputated and in those in whom examining their body composition is difficult; those with low body weight (≤40 kg); and pregnant women.

### 2.6 Randomization and blinding

Computer-based block randomization will be employed to ensure an equal number of participants. In MODE phase I (MODE-I), participants will be randomly assigned in a ratio of 1:1 to either the experimental group (app-delivered exercise group) or active control group (booklet-delivered exercise group). Both groups will perform 3-month self-exercise. In MODE phase II (MODE-II), participants who complete the 9-month self-exercise in each group will be randomly assigned in a ratio of 1:1 to the experimental group (feedback+ app group) or active control group (feedback– app group). Allocation will be concealed using sequentially numbered opaque envelopes. These envelopes will be opened after obtaining consent for trial participation. A random assignment number will then be recorded in the case record form (CRF), and the study will be performed according to the assigned group. Because of the characteristics of this test method, the outcome assessor will also be blinded to the group allocation. Once the intervention is completed, the researcher responsible for statistical analysis will receive an Excel (Microsoft, Redmond, WA) spreadsheet with the necessary data without any identifiable parameters. A flow diagram of the study is shown in Figure 1.

### 2.7 Study interventions

#### 2.7.1 MODE-I

**Figure 2:**
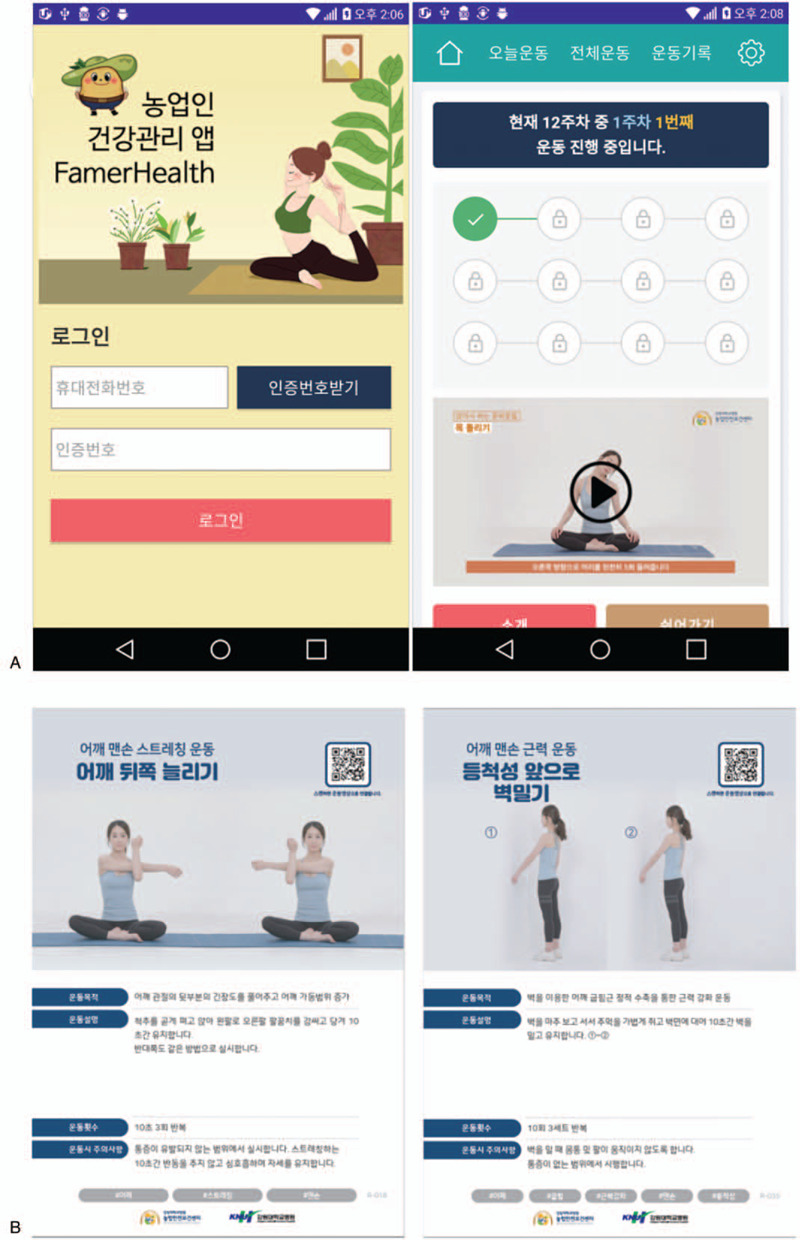
Mobile app (A) and booklet (B) for the self-exercise program.

Participants who meet the inclusion criteria will be randomized to the app or booklet. Both groups will receive an individual-tailored exercise program via the mobile app or booklet (Fig. 2).

##### 2.7.1.1 Individual-tailored exercise program

Initially, the shoulders, knees, and low back will be evaluated, and musculoskeletal health status for each region will be divided into 3 levels to provide individualized exercise programs. Musculoskeletal function assessment includes self-administered questionnaires (Shoulder Pain and Disability Index [SPADI], Western Ontario and McMaster Universities [WOMAC], and Oswestry Disability Index [ODI]) and are categorized as mild and severe groups for each part (shoulder, knee, and back). The exercise for each part is programmed for 3 months with 4 levels of difficulty (from level 1 with the lowest difficulty to level 4 with the highest difficulty) (Table 2). Each exercise level for shoulder, knee, and back will be prescribed differently depending on their musculoskeletal health status, respectively. Level 1 is prescribed for high risk and level 2 for moderate risk for MODE-I (Table 3).

**Table 2:** Levels of exercise for the shoulder, knee, and low back.

	Shoulder	Knee	Low back
Level ½	Trunk and shoulder stretchingIsometric shoulder forward/abduction/external-rotation exerciseScapular stabilizing exercise	Quadriceps exerciseStep up	Bird-dog easyAbdominal exercise easySide-bridging exercise easy
Level 3	Isotonic exercise using elastic band(s)	Lunge	Supine bridging
Level 4	Isotonic diagonal exercise	Balance	Plank

**Table 3:** Overview of the content of the self-exercise plans.

Risk levels	MODE phase I	MODE phase II (II-1)	MODE phase II (II-2)	MODE phase II (II-3)
Mild	Level 2	Level 2 or 3	Level 2, 3, or 4	Level 2, 3, or 4
High	Level 1	Level 1 or 2	Level 1, 2, or 3	Level 1, 2, 3, or 4

The exercise characteristics include the following: type: 1 stretching and 1 strengthening exercises for the shoulder, knee, and back (total 6 exercises per day); frequency: 3 times per week; duration: 20 min/day; and intensity: 2 levels (Table 2).

##### 2.7.1.2 Experimental group

The experimental group (app-delivered exercise group) will perform 3-month self-exercise using a smartphone app.

##### 2.7.1.3 Active control group

Subjects belonging to the active control group (booklet-delivered exercise group) will perform 3-month self-exercise provided by booklets.

#### 2.7.2 MODE-II

##### 2.7.2.1 Individual-tailored exercise program

The following outcomes, including exercise completion, will be assessed at 3 months, and the subjects will be randomly allocated to MODE-II groups. The difficulty level of the exercise program will be adjusted individually at the time of allocation to MODE-II. Every 3 months, the level and the level of the exercise program will be evaluated and the level of difficulty will be changed (Table 3).

##### 2.7.2.2 Experimental group

In MODE-II, the experimental group (feedback+ app group) will exercise using a smartphone app that adds feedback to the subjects (Table 4).

**Table 4:** Real-time feedback provided by the mobile application.

Types of feedback	Schedule reminder	Real-time feedback	Get badge
Explanation	Reminder of the day's exercise schedule	Notification of the progress of exercise and comparison with the exercise yesterday	Award badge for successive workouts
Example	1) “Today is a workout day.”2) “An exercise reservation is made at 7 pm today.”	1) After 10 minutes of stopping the exercise video“You achieved 50% of today's workout. Try a little harder”“You have achieved 100% of today's workout. Awesome! Keep exercising like you have just been doing”2) When the specified (alarm) exercise time is over 30 min“You would have achieved the exercise goal if you…”“What are you doing? Start again with a new resolution and work harder”	1) Achievement of workouts (1, 3, 5, 7, 10, 15, 20, 25, 30, and 100 workouts)2) Continuous recording (2, 3, 4, 5, 6, and 7 wk; 2, 3, 4, 5, 6, 7, 8, 9, 10, and 11 mo; and 1 y)3) Weekly exercise (3 times a wk, 2 times a wk, 4 times a wk twice, 5 times a wk, 6 times a wk, 7 times a wk)4) By exercise time (early bird/owl)

##### 2.7.2.3 Active control group

Subjects belonging to the active control group will perform self-exercise using a smartphone app without the feedback function (feedback- app group).

### 2.8 Assessment

The primary and secondary outcomes will be assessed at baseline, 3 months, 6 months, 9 months, 12 months, and postintervention (Table 1). All participants will be requested to complete follow-up assessments, except for those who withdraw or are withdrawn from the study. Those who withdraw will be asked to identify reasons for withdrawal. After the completion of the MODE-II exercise, all subjects will undergo close-out assessment.

### 2.9 Baseline assessment at baseline and close-out

Blood pressure, pulse rate, and waist circumference will be measured. Bone density will be assessed using dual-energy x-ray absorptiometry. Respiratory evaluations including chest radiography and pulmonary function test will be conducted, in addition to ophthalmic examination including tonometry and funduscopy. Laboratory tests will be conducted including total cholesterol, low density lipoprotein-cholesterol, high-density lipoprotein-cholesterol, triglyceride, blood urea nitrogen, creatinine, aspartate aminotransferase, alanine aminotransferase, fasting glucose, and hemoglobin A1c.

### 2.10 Primary outcomes

The completion rate of the self-exercise program at the completion of MODE-I (3 months) and MODE-II (12 months) will be assessed with phone call assessment. Participants will be asked about the self-exercise as follows: “Did you exercise using the app (or booklet) we provided in the past 3 months?”

### 2.11 Secondary outcomes

#### 2.11.1 Maintaining self-exercise with MODE-II

Maintenance rate of self-exercise program every 3 months during MODE-II (6 and 9 months) will be assessed with phone call assessment. Exercise time using the app will be assessed and compared between the experimental and control groups in MODE-II.

#### 2.11.2 Musculoskeletal evaluations

Participants will be assessed for musculoskeletal pain and disability. Physical examinations and radiography for the shoulder, knee, and lumbar spine will be conducted at baseline and at close-out. Musculoskeletal pain and disability will be assessed using the SPADI,^[6]^ WOMAC,^[7]^ and ODI.^[8]^ Physical evaluations will be conducted, including range of motion and tenderness evaluations and the Neer test for the shoulder joints; range of motion and tenderness evaluations for the knee joints; and the straight leg raising test and foot dorsum sensory, long toe extensor weakness, and tenderness evaluations for the lumbar spine. Radiography for both knees (Rosenberg view), lumbar spine (anteroposterior and lateral view), and both hands (posteroanterior view) will be conducted.

The SPADI was developed for the assessment of patients with shoulder pain.^[6]^ The SPADI was translated into Korean, and its validity and reliability were assessed.^[9]^ The SPADI is a self-administered questionnaire consisting of 13 items divided into 2 subscales: a 5-item subscale that measures pain (pain symptoms) and an 8-item subscale that measures disability (physical function). The participant is instructed to place a mark on the numerical rating scale (NRS) ranging from 0 (best) to 10 (worst) for each item that best represented their shoulder problem. Each subscale is summed and transformed to a score out of 100, with a higher score indicating greater impairment or disability.

The WOMAC was developed specifically to measure the outcomes of patients with lower extremity arthritis. The WOMAC was translated into Korean, and its validity and reliability were assessed.^[7]^ The WOMAC is a self-administered questionnaire and contains 24 items categorized in 3 subscales: pain, stiffness, and physical function. Each item is scored as 5-point Likert scale format: none (0), mild (1), moderate (2), severe (3), and extreme (4). Scores for each subscale were determined by summing the component item scores for each subscale (possible score range, Pain: 0–20, Stiffness: 0–8, Physical function: 0–68). The final total aggregated scores (possible score range: 0–96) were determined by summing the scores for each subscale. ODI is a self-administered 10-item questionnaire covering intensity of pain and disability in standing, personal care, sleeping, lifting, sex life, walking, social life, sitting, or traveling. ODI was translated into Korean, and its validity and reliability were assessed.^[10]^ Scores for each item range from 0 (no pain or disability) to 5 (severe pain or dis- ability). Scores are reported as percentages according to the following formula: total score (0–50)/maximum score (50) × 100.^[8]^

#### 2.11.3 Muscular strength

The muscular strength of the trunk extension and hand grip will be evaluated during the baseline and close-out assessments.

Isometric trunk extensor strength will be measured in the sitting posture using a hand-held dynamometer (Power Track II Commander Muscle Tester, JTECH Medical, UT) positioned at the inferior angle of the scapula (at the level of T7). A custom-made chair designed by authors will be used, which ensures that participants’ feet are off the floor to remove the influence of the lower limbs.^[11]^

Hand grip strength will be assessed by trained technicians using a hydraulic hand dynamometer (SH5001, Saehand Corp., Masan, Korea). Participants will be seated comfortably on a chair without armrests, and the shoulder will be adducted and neutrally rotated, the elbow will be held at 90° of flexion, and the forearm and wrist will be maintained in a neutral position.

#### 2.11.4 Balance ability

The Timed Up & Go (TUG) is a test of balance. Participants will be required to sit on a chair, stand and walk a 3-m course at a comfortable speed, walk back to the chair and sit again. The time from standing up to sitting down again will be measured, with prolonged time associated with increased falling risk.^[12]^

The Berg Balance Scale (BBS) is an evaluation tool designed to measure balance and is composed of 14 items.^[13]^ Each item is made up of 5-point scale ranging from 0 to 4, with 0 indicating the lowest level of function and 4 the highest level of function. The BBS was translated into Korean, and its reliability was assessed.^[14]^ The total possible score on the BBS is 56, which indicates excellent balance. A score of <45 indicates individuals that have a greater risk of falling.^[15]^

#### 2.11.5 Muscle mass and fat mass

Muscle mass and fat mass will be measured with a body composition analyser (Inbody370S, Seoul, Korea) using direct segmental multi-frequency bioelectrical impedance analysis at baseline and close-out.

#### 2.11.6 Depression

The Patient Health Questionnaire-9 (PHQ-9) is a self-administered questionnaire and is a valuable screening instrument for detecting major depressive disorders.^[16]^ PHQ-9 was translated into Korean, and its validity and reliability were assessed.^[17]^ PHQ-9 includes 9 items pertaining to the Diagnostic and Statistical Manual of Mental Disorders-IV criteria for major depressive disorder: anhedonia; depressed mood; trouble sleeping; feeling tired; change in appetite; guilt, self-blame, or worthlessness; trouble concentrating; feeling slowed down or restless; and thoughts of being better off dead or hurting oneself.^[16]^ Each item is rated on a 4-point scale from 0 to 3 (0—never; 1—several days; 2—more than half the time; and 3—nearly every day) during the 2 weeks before and including the day of survey completion. The overall scores range from 0 to 27. A cutoff score of 5 is associated with a sensitivity of 80% and specificity of 78% for depressive disorders.^[17]^

### 2.12 Criteria for discontinuation

Voluntary withdrawal of consent from the trial.

### 2.13 Harms and benefits

Each exercise is commonly used. All exercises and using the application are not expected to be inconvenient or unsafe to the user. If there is a condition that requires the termination of the research due to medical or subjective reasons, the corresponding patient will be withdrawn from the study. Reasons for discontinuation will be recorded.

### 2.14 Data and Safety Monitoring Plan

Each participant's identification number and baseline data will be contained in CRFs. Outcome data will be recorded in the CRF by 2 independent trained research assistants with a double-entry method. Data on assessments will be monitored. Only those who have been previously authorized by the principal investigator can access the data. The data monitoring committee will check the state of recruitment, assessed data, and adverse events, and will guide the research team regarding any needed steps.

### 2.15 Sample size calculation

We assumed that the exercise completion rate with 3-month self-exercise using the smartphone app would be 60%; we expected that the completion rate with 3-month self-exercise by providing physical education data (booklets) would be 40%. With these assumptions, we estimated that we would need to enroll 200 participants for MODE phase I with an alpha level of 0.05 and a beta level of 0.20. With 20% of phase I subjects expected to drop out, 160 subjects would be needed for MODE phase II.

### 2.16 Statistical analysis

Statistical analyses will be performed using SPSS version 24 (IBM, Armonk, NY). We will use the independent samples *t* test (Student *t* test) to evaluate quantitative data with a normal distribution and the Mann-Whitney *U* test to evaluate data without a normal distribution. The Pearson *χ*^2^ test or Fisher exact test will be used to analyze qualitative comparative data. *P* values of <.05 will be considered significant.

### 2.17 Patient and public involvement

Patients or the public were not involved in the design of this study.

### 2.18 Ethics and dissemination

The study protocol has been approved by the research ethics committee of the Kangwon National University Hospital Institutional Review Board (KNUH-2020-03-005-003) and was registered at the Clinical Research Information Service of Korean National Institutes of Health (KCT0005245) on July 17, 2020. All subjects will be provided with the details of the study and they will provide written informed consent before participation.

## 3 Discussion

This will be the first attempt to compare methods using booklets and apps to identify effective ways of providing personalized self-exercise programs according to musculoskeletal health stages via the examination of female farmers (MODE-I). With this study, it will be possible to confirm whether the mobile app is effective in the maintenance and completion of self-exercise compared to using a conventional booklet. We will conduct a MODE-II study to assess the effect of adding a feedback function to the mobile app. Finally, we will evaluate the health status of the musculoskeletal system according to the degree of participation in the self-exercise program for 12 months to confirm the effect of self-exercise. This may be a useful method for managing musculoskeletal health in farmers living in rural areas, and it may be a method for promoting health in the “untact” era.

### 3.1 Related articles

No publications containing the results of this study have been published or submitted to any journal.

## Acknowledgments

We acknowledge the collaborators working at the Kangwon National University Hospital.

## Author contributions

SB, GK, and HP conceived the study and contributed to the planning of the study and were major contributors regarding writing the manuscript, reading and revising the manuscript, and the statistical analysis plan. GK will be responsible for the statistical analyses. SB will conduct the data collection. The authors read and approved the final manuscript.

**Conceptualization:** Sora Baek, Gowun Kim, Hee-won Park.

**Data curation:** Sora Baek.

**Formal analysis:** Gowun Kim.

**Funding acquisition:** Sora Baek.

**Methodology:** Sora Baek, Gowun Kim.

**Visualization:** Sora Baek, Gowun Kim, Hee-won Park.

**Writing – original draft:** Sora Baek, Gowun Kim, Hee-won Park.

**Writing – review & editing:** Sora Baek, Gowun Kim, Hee-won Park.
